# Dynamic left ventricular outflow tract obstruction complicating aortic valve replacement: A hidden malefactor revisited

**DOI:** 10.4103/1658-354X.65118

**Published:** 2010

**Authors:** Prashanth Panduranga, Madan Mohan Maddali, Mohammed Khamis Mukhaini, John Valliattu

**Affiliations:** Department of Cardiology, Royal Hospital, Muscat, Sultanate of Oman; 1 Department of Cardiology, Royal Hospital, Muscat, Sultanate of Oman; 2 Department of Cardiothora Surgery, Royal Hospital, Muscat, Sultanate of Oman

**Keywords:** *Aortic valve replacement*, *dynamic left ventricular outflow tract obstruction*, *cardiogenic shock*

## Abstract

It is known that a dynamic left ventricular outflow tract (LVOT) obstruction exists in patients, following aortic valve replacement (AVR) and is usually considered to be benign. We present a patient with dynamic LVOT obstruction following AVR, who developed refractory cardiogenic shock and expired inspite of various treatment strategies. This phenomenon must be diagnosed early and should be considered as a serious and potentially fatal complication following AVR. The possible mechanisms and treatment options are reviewed.

## INTRODUCTION

It is known that dynamic left ventricular outflow tract (LVOT) obstruction exists in patients following aortic valve replacement (AVR).[1–7] Aurigemma *et al*.[1] reported that the presence of dynamic LVOT obstruction is associated with increased mortality. On the contrary, Bartunek *et al*.[2] concluded that dynamic LVOT obstruction is associated with high in-hospital morbidity but excellent early and long-term survival. We present a patient with dynamic LVOT obstruction following AVR, who developed refractory cardiogenic shock and expired inspite of various treatment modalities. The possible mechanisms and treatment options are discussed.

## CASE REPORT

A 65-year-old man, hypertensive with a history of percutaneous coronary intervention to left anterior descending artery and exertional dyspnea, was referred for AVR. Transthoracic echocardiography demonstrated a calcified aortic valve with peak and mean gradients of 80 and 45 mmHg, respectively. The left ventricular (LV) end-diastolic and systolic diameters were 44 and 25 mm, respectively, with an EF of 76%. There was severe concentric LV hypertrophy with no systolic anterior motion (SAM) of mitral valve and no dynamic LVOT obstruction. Basal septum measured 21 mm in diastole and 24 mm in systole, posterior wall measured 18 mm in diastole and 21 mm in systole, and LVOT measured 21 mm. E/A wave ratio in mitral inflow showed impaired relaxation pattern. The coronary stent was patent at angiography with tight ostial stenosis of second diagonal vessel. At surgery, left internal mammary artery was grafted to diagonal and his stenotic aortic valve was replaced with a 21-mm Carpentier-Edwards Perimount aortic heart valve under general anesthesia, normothermic cardiopulmonary bypass (CPB) and antegrade cold blood cardioplegia. CPB and aortic cross clamp times were 100 and 70 minutes, respectively. The patient had a body surface area of 1.58 m^2^ and the calculated indexed effective orifice area of prosthesis was 0.82 cm^2^/m^2^.

**Figure 1:**
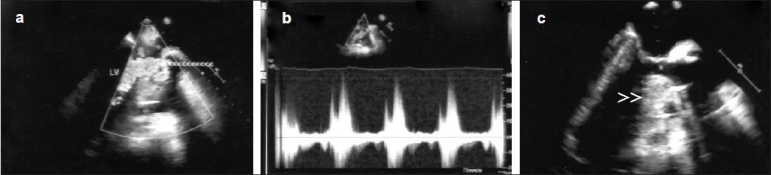
Intraoperative transesophageal echocardiogram following AVR showing turbulent abnormal flow in the LVOT (a, arrow heads) with a dynamic peak gradient of 58 mmHg (b) and a small left ventricle with thick basal sigmoid shaped septum (c, arrow heads) causing narrow LVOT; LV, left ventricle

At the time of separation from CPB, the patient developed profound hypotension (systolic/diastolic blood pressure: 60/40 mmHg) which did not respond to incremental doses of vasopressors (epinephrine, norepinephrine) and to intraaortic balloon pump (IABP) counter pulsations. There were no ischemic changes noticed in the monitor. An emergency transesophageal echocardiogram was performed which showed a small hypercontractile LV and an abnormal turbulent flow velocity at the LVOT [Figure 1a, arrow heads] with peak and mean systolic gradients of 58 and 39 mmHg, respectively [Figure 1b]. Peak and mean gradients across the prosthetic aortic valve were 17 and 8 mmHg, respectively. There was no SAM or midcavity obstruction noted but the basal septum was very thick and sigmoid shaped causing a turbulent flow at the LVOT [Figure 1c, arrow heads]. Mitral/aortic valve and right ventricle were normal. A diagnosis of dynamic LVOT obstruction was made and IABP was discontinued. A subaortic limited septal resection was performed as the thick septum was thought to be the cause for dynamic gradient. The hemodynamic readings were: mean systemic arterial pressure 50 mmHg; mean pulmonary artery pressure 20 mmHg; pulmonary capillary wedge pressure 15 mmHg; right atrial pressure 9 mmHg; cardiac index 2.0 L/min/m^2^; and systemic vascular resistance index 1638 dynes s/cm^5^m^2^. Phenylephrine boluses along with infusion were initiated to increase systemic vascular resistance, norepinephrine was continued, and the patient was separated from CPB after optimization of preload. A pulmonary capillary wedge pressure of 20–25 mmHg was aimed at. Atrioventricular synchrony was maintained with the help of atrioventricular pacing (DDD) at a heart rate of 60 beats per minute. The patient was shifted to the postcardiac surgical unit without sternal approximation with a systolic blood pressure of 80 mmHg. There was persistence of LVOT gradient. Despite resuscitative measures, patient went into refractory shock and finally developed pulseless electrical activity and expired.

## DISCUSSION

In patients undergoing AVR, there are two sites where dynamic intraventricular gradient exists, the LVOT and midventricular area.[1,2] The mechanisms described are either muscular cavity obliteration or SAM.[1,2] In patients with dynamic LVOT obstruction without SAM, the obstruction is due to LV hypertrophy especially involving basal septum resulting in a narrow LVOT.[4] After AVR, there is a fall in the end systolic LVOT pressure following relief of downstream obstruction (which was holding the walls apart) leading to apposition of already narrowed LVOT walls during systole and exacerbating obstruction. It is thought that the removal of a fixed obstruction will “unmask” dynamic obstruction, as LV end-systolic pressure falls.[5] In addition to this, physiological factors like filling state, contractility, systemic vascular resistance (post-CPB distributive shock may contribute), and small LV volume (after AVR) determine whether more severe obstruction occurs. This leads to flow acceleration and abnormal gradient which are epiphenomena of the hyperdynamic state in an extremely small cavity and reflect abnormal ejection dynamics.

The preoperative echocardiographic factors associated with the dynamic LVOT obstruction[1–4] are small ventricular diameters, high transvalvular gradient, good overall contractility, discrete asymmetric hypertrophy, sigmoid shaped ventricular septum,[6] and tendency to a small LVOT. All these factors were present in our patient. In post-AVR patient-prosthesis mismatch (PPM), flow acceleration begins at the level of the prosthesis, whereas flow acceleration and turbulence are evident in the LVOT in patients with dynamic LVOT obstruction as seen in our patient.[4] Our patient had moderate PPM. In a study using the same valve as ours in 506 patients, moderate PPM (iEOA > 0.65 and < 0.85 cm^2^/m^2^) was not an independent predictor of early mortality.[8]

Management of dynamic LVOT obstruction post-AVR is complex. In patients who are hemodynamically stable, the treatment is to alter physiological conditions exacerbating obstruction. This involves filling, reduction in contractility with beta-blockers, andafterload increase with vasoconstrictors (α_1_ agonists – phenylephrine). Beta-blockers decrease force of ventricular contraction and ventricular ejection acceleration, thus reducing SAM of the mitral valve (if present), aortic outflow obstruction, and the final aortic pressure gradient.[9] Another benefit of β-blockers is their effect in decreasing heart rate; the decrease can increase ventricular preload by facilitating greater ventricular relaxation and longerfilling before ventricular ejection. α_1_ -Agonists increase the size of the functional out-flow tract and decrease the LVOT pressure gradient by increasing systemic vascular resistance and end-systolic and end-diastolic left ventricular volume.[10] Inotropes (β-agonists/milrinone) or IABP may worsen the condition by decreasing afterload;[11] hence, they were discontinued in our patient once the diagnosis was suspected. When beta-blockers cannot be used due to shock, dual chamber pacing with low heart rate can be used in reducing heart rate and contractility with subsequent reduction in LVOT gradient.[12] In patients demonstrating SAM, either mitral valve repair or replacement is done.[5] Other treatment option described is prophylactic myectomy during surgery for patients with marked septal hypertrophy.[1,4,5–6] However, in patients who are hemodynamically unstable, a cautious application of the above measures need to be taken as in our patient, even though the final result was not encouraging. In conclusion, the presence of dynamic LVOT obstruction following AVR must be diagnosed early and should be considered as a potentially fatal complication which may be refractory to treatment and catastrophic.
